# Sustainable Approach to Prolong Cold Storage Shelf Life of Plant-Based Meat Using Lactic Acid Bacteria

**DOI:** 10.3390/foods14223923

**Published:** 2025-11-17

**Authors:** Khemmapas Treesuwan, Kullanart Tongkhao, Hataichanok Kantrong, Kanokwan Yodin, Jutamat Klinsoda, Pathika Pengpinit

**Affiliations:** 1 Institute of Food Research and Product Development, Kasetsart University, Bangkok 10900, Thailand; ifrhnk@ku.ac.th (H.K.); ifrkwd@ku.ac.th (K.Y.); ifrjmk@ku.ac.th (J.K.); ifrptkk@ku.ac.th (P.P.); 2 Department of Food Science and Technology, Faculty of Agro-Industry, Kasetsart University, Bangkok 10900, Thailand

**Keywords:** sustainability, biological preservative, clean label, high-moisture extrusion, amino acid score

## Abstract

The growing global population has highlighted the need to replace animal-based meat with plant-based meat (PBM) as a protein source. Using lactic acid bacteria (LAB) offers a promising and sustainable approach to prolong PBM shelf life and maintain quality comparable to non-food additives. This study investigated the potential of LAB to improve the qualities of PBM products. Three LAB strains, *Lactiplantibacillus plantarum* (LM), *Lactiplantibacillus pentosus* (LS), and *Pediococcus acidilactici* (PA) were selected from vegetable sources, and their effects on PBM shelf life were monitored for 21 days at 4 °C. Results showed that PBM samples treated with both *Lactiplantibacillus* spp. maintained consistent color properties throughout the cold storage period. Textural analysis revealed that the control samples exhibited the lowest hardness, springiness, gumminess, and chewiness, while LS-treated samples showed the highest values. Both *Lactiplantibacillus* spp. treated samples had pH values at less than 5, with no statistically significant differences. Volatile organic compounds were not impacted by LAB. LM-treated PBM exhibited higher amino acid content compared to LS and non-LAB-treated samples. Our findings showed that *L. plantarum* improved the texture and prolonged the shelf life of PBM products at 4 °C for 21 days. Results indicated that *L. plantarum* could be used as an alternative sustainable green biological preservative agent, serving as a clean label product.

## 1. Introduction

Plant-based meat (PBM) contributes to environmentally friendly (green) sustainable food production by addressing the global imbalance between meat supply and demand [1,2,3,4]. Nowadays, people associate healthy foods with lower additives and chemical substances, termed “clean label” products [5]. This trend has led to the use of natural preservatives instead of synthetic chemicals to improve the microbiological quality of foods [6]. Antimicrobial agents (AAs) are chemical or natural compounds that extend the shelf life of food commodities by inhibiting the growth or eliminating spoilage microorganisms [7,8]. AAs of microbial origin have recently gained credibility as a natural, renewable source.

High-moisture PBM is formed using high-moisture extrusion technology (above 40% moisture) [9] to create a plant-based product with a structure and texture resembling meat [10]. Guyony et al. [11] found that high-moisture extrusion produced meat-like textures through the creation of a fibrous structure with thin layers. This technology develops a PBM comparable to the fibrous structure of meat [12]. PBM was produced, using a high-moisture extruder, from 50% defatted soy flour, 20% soy protein isolate, and 30% wheat gluten, with results showing a desirable meat-like structure [13].

Factors limiting public acceptance of PBM include nutrition, taste, texture, short shelf life, and flavor (mown grass odor) [14]. Flavor is a critical limiting factor in the acceptability and marketability of PBM. Undesirable chemosensory perceptions, such as bitter taste and astringency, impact consumer acceptance [15]. The molecular and physicochemical properties of plants (mung bean and soybean), lower protein efficiency, and lack of nutrients compared with meat contribute to these limitations [14]. The processing steps of soy, wheat, and pea proteins impact the occurrence, survival, and growth of spoilage and pathogenic microorganisms in PBM products [16,17,18]. Roch et al. [19] found that different spoilage profiles depended on raw material composition and environmental conditions during storage. Barmettler et al. [16] reported the total viable count of live, culturable microorganisms before the expiry date as <2–7 log CFU/g in PBM products collected from retail outlets in Switzerland. Various chemically synthesized reagents have been used as food additives to control spoilage and prevent microbial contamination [20]. Modern consumers are becoming increasingly concerned about the potential health risks of using chemicals as food additives [21]. When fermented, natural antibacterial agents such as lactic acid bacteria (LAB), generally regarded as safe (GRAS), produce beneficial metabolites such as organic acids, lipids, and bacteriocin-like substances, which inhibit the growth and reproduction of foodborne pathogens [7]. Du et al. [22] reported that the addition of LAB prolonged the shelf life of foods, while [23] noted that LAB addition improved the unpleasant beany flavor in soybean.

Microorganisms have long been used for preserving food and enhancing its nutritional value and sensory qualities, including appearance, aroma, texture, and taste [24], with different microorganisms used to produce AAs. Among these, probiotics are particularly noteworthy due to their capacity to modulate the microbiota of humans and foods. Probiotics have recognized health benefits and reduce the risks of infection [25]. Probiotic microorganisms include LAB, a heterogeneous group of Gram-positive and catalase-negative microorganisms. LAB comprise genera such as *Lactiplantibacillus*, *Lactococcus*, and *Pediococcus*; they serve as natural food preservatives by producing organic acids and antimicrobial metabolites, which inhibit spoilage and pathogenic microbes [26]. Soy-based plant products fermented with LAB showed reduced bitterness and beany off-flavors [27]. Using LAB is a low-cost method for food preservation, effectively inhibiting pathogens such as *Listeria*, *Clostridium*, *Staphylococcus*, *Bacillus*, *Aspergillus*, and *Penicillium* [21,28,29]. Since strains of *Lactiplantibacillus plantarum* [21,30,31], *L. pentosus* [32,33,34,35], and *Pediococcus acidilactici* [36] demonstrated inhibitory effects against a variety of pathogens of concern in PBM, applying these bacteria could prevent quality deterioration. This study investigated three LAB strains, *Lactiplantibacillus plantarum* (LM), *L. pentosus* (LS), and *Pediococcus acidilactici* (PA) in PBM products, and evaluated the microbial qualities, physicochemical characteristics, volatile organic compounds, and amino acid profiles of PBM products stored at 4 °C for 21 days. Objective of this study was to provide an alternative approach to improve PBM qualities and prolong its shelf-life compared to non-LAB treated PBM.

## 2. Materials and Methods

### 2.1. Raw Materials

Mung bean protein isolate (MPI), 80% protein content, was purchased from ADVANTEC Co., Ltd., Pathum Thani, Thailand. Wheat gluten (WG) > 82% protein content was obtained from Krungthepchemi Co., Ltd., Bangkok, Thailand. Defatted soy flour (DFSF) was obtained from Omshree Agro Tech Co., Ltd., Dhule, India. The three raw materials were mixed in the ratio MPI:WG:DFSF as 2:3:5 (dry basis) following [13] with slight modifications by replacing soy protein with mungbean protein.

### 2.2. PBM Product Preparation

**Figure 1:**
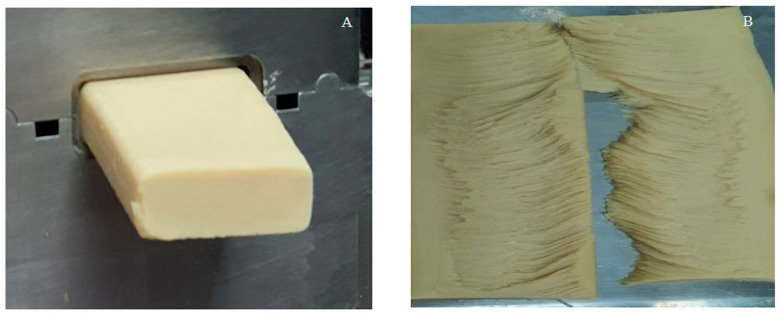
Appearance of PBM: (**A**) after production by a high moisture extruder and (**B**) after tearing.

PBM was prepared according to [37] with some modifications. The PBM product was processed using a co-rotating twin screw extruder (Coperion ZSK 18 Megalab, Coperion GmbH, Stuttgart, Germany). The MPI/WG/DFSF mixture was fed into the extruder at a constant speed of 6 kg/h (dry basis), with water injected at the second barrel by a volumetric pump (Watson Marlow, Falmouth, UK). The temperatures of barrel zones 1–9 were 25, 50, 80, 80, 100, 120, 130, 120, and 100 °C. A rectangular die with internal dimensions of 50 × 15 × 800 mm (W × H × L) was connected to the end of the extruder barrel (Figure 1A). The die temperature was 60 °C. The extrudate or PBM product was cut at the end of the cooling die into pieces 50 × 15 × 200 mm (W × H × L). The torn PBM fibrous structure is shown in Figure 1B. The PBM products were kept at 4 °C during further investigation of the quality of the control samples (non-LAB-treated) and artificial PBM samples (LAB-treated), inoculated with lactic acid microorganisms *L. plantarum* (LM), *L. pentosus* (LS), and *Pediococcus acidilactici* (PA) throughout the 21 days of storage.

### 2.3. Lactic Acid Bacteria Preparation

LAB (*Lactiplantibacillus plantarum*, *Lactiplantibacillus pentosus*, and *Pediococcus acidilactici*) were isolated from fermented vegetables by culture-dependent methods, 16S rRNA sequencing and Matrix Assisted Laser Desorption/Ionization Time of Flight (MALDI-TOF) (Microflex LT bench-top, Bruker Daltonics, Bremen, Germany) [38]. The three strains were grown in de Man Rogosa and Sharpe broth (MRSB; Merck, Darmstadt, Germany) at 37 °C for two successive 24 h and 18 h transfers before use. The pellet and supernatant were separated by centrifugation (Sorvall, RC 6 Plus, Thermo Fisher Scientific, NC 28804, Waltham, MA, USA) at 10,000 rpm for 10 min and the pellets were washed in sterile 0.85% NaCl. The pellet from each strain was washed twice with sterile 0.85% NaCl and suspended in this solution to a final cell density concentration of 8 log CFU/mL of stock inoculum [39].

### 2.4. PBM Sample Preparation and Storage

A 5 mL aliquot of each LAB strain of *L. plantarum*, *L. pentosus*, and *P. acidilactici* was added to 45 mL of 0.85% NaCl. The LAB suspension was then added to 450 g of PBM in a nylon bag and hermetically sealed, with an initial load of LAB at 5 log CFU/g [40]. The initial counts of the three LAB strains were determined by plating on de Man Rogosa and Sharpe agar (MRS; Merck, Darmstadt, Germany) before setting up following their inoculation protocol into the PBM product. Subsequently, the LAB count in the product was quantified by plating again on MRS agar after packing and before incubation. The three PBM products with LAB samples were incubated at 37 °C for 48 h as the 0-day sample. A non-LAB-treated sample as the control was packed and hermetically sealed in a nylon bag with 50 mL of 0.85% NaCl and 450 g of PBM. All the samples were stored at 4 °C for 21 days. PBM qualities were determined by randomly selecting three samples from each treatment every 7 days for 21 days.

### 2.5. Microbial Analysis of PBM Samples

For the microorganism analyses, 10 g of the three subsamples, randomly taken from each treatment and storage condition, were combined with 90 mL of 0.85% NaCl and homogenized in a stomacher (BagMixer 400, CC, St. Nom, France) for 1 min [39]. The homogenized samples were then diluted in 0.85% NaCl and plated on the following media (using 100 µL of each dilution per plate in duplicate): (I) plate count agar (PCA; Merck, Darmstadt, Germany) for total plate counts, incubated at 37 °C for 48 h, (II) potato dextrose agar (PDA; Merck, Darmstadt, Germany) for total yeasts and molds, incubated at 30 °C for 72 h, (III) de Man Rogosa and Sharpe agar (MRS; Merck, Darmstadt, Germany) for LAB incubated at 37 °C for 48 h, and (IV) mannitol egg yolk polymyxin agar (MYP; Merck, Darmstadt, Germany) for total MYP count, incubated at 30 °C for 24 h.

### 2.6. pH Value and Titratable Acidity

One gram of the LAB-treated PBM samples and the non-treated PBM samples was immersed in 10 mL of deionized water and homogenized for 1 min [41]. The pH of the mixture was measured using a pH meter (Oakton pH 700 Benchtop Meter, Cole-Parmer, Vernon Hills, IL, USA). Titratable acidity (TA) was determined following a modified version of the method described by [42]. Five grams of PBM were mixed with 45 mL of distilled water and titrated with 1.0 N sodium hydroxide (NaOH) using phenolphthalein as an indicator [43]. The TA was expressed as the equivalent lactic acid content and calculated using the equation:

(1) $$\%\mathrm{TA}(\mathrm{as}\mathrm{lactic}\mathrm{acid})=\frac{V_{\mathrm{NaOH}}{\;\times \;M}_{\mathrm{NaOH}}{\;\times \;\mathrm{MW}}_{\mathrm{lactic}}\;\times \;100}{V_{s}\;\times \;100}$$

where V_NaOH_ represents the volume of NaOH (mL), M_NaOH_ represents the normality of NaOH, MW_lactic_ represents the molecular weight of lactic acid (C_3_H_6_O_3_) = 90.08 g/mol, and V_s_ represents the volume of the sample according to [43].

### 2.7. Determination of Volatile Organic Compounds (VOCs) by HS-SPME/GC-MS

The VOCs of PBM were analyzed using HS-SPME/GC-MS (GC-8890 & MS-5977c, Agilent Technologies, Santa Clara, CA, USA) following the method of Wellington da Silva Oliveira [44] with minor modifications. Briefly, 3 g of mashed sample were added to 1 µL of internal standard, 1 g of NaCl, 3 mL of DDI water, and a stir bar in a 20 mL vial. The sample vial cap was sealed with septa and incubated for 15 min at 45 °C, 250 rpm. The SPME extraction head was inserted into the vial and adsorbed for 45 min at 45 °C, 250 rpm with constant headspace. The extraction head was then inserted into the GC-MS instrument and the solution was dissolved at 260 °C for 5 min. The temperature program started with a column temperature of 35 °C, held for 5 min, then increased to 225 °C at 4 °C/min and held for 10 min. The carrier gas was helium with a flow rate of 1 mL/min.

### 2.8. Physicochemical Characteristics of the PBM Products

#### 2.8.1. Color Analysis of the PBM Products

Color measurements of the LAB-treated PBM samples and the non-treated PBM samples (control) were performed using a spectrophotometer (Spectraflash 600 plus, Datacolor International Co., Lawrenceville, NJ, USA) according to the CIELAB 1976 color space. Ten random measurements were conducted on each sample to assess L* (lightness, ranging from black to white), a* (red–green axis), and b* (yellow–blue axis). Chromatic aberration was quantified following the equation [45]:

∆E = [(∆L*)^2^ + (∆a*)^2^ + (∆b*)^2^]^0.5^ (2)

Ten independent measurements (n = 10) were conducted for each PBM sample to ensure statistical robustness [46].

#### 2.8.2. Textural Analysis

The textural properties of the PBM samples were evaluated using a Texture Analyzer (Model TA-XT2i, Stable Micro Systems Ltd., Godalming, UK) at room temperature. A two-cycle texture profile analysis (TPA) compression test was performed to determine hardness (force required for initial chewing), resilience (elasticity measured by resistance after the first pressing), cohesiveness (textural characteristic indicative of food adhesion), springiness (elasticity of food that returns to its original shape when pressed), gumminess (characteristic of semi-solid food that breaks apart and is ready to swallow), and chewiness (resistance to chewing) following the modified protocol of [23]. The PBM samples were cut into 1.5 × 1.5 × 1.5 cm (W × L × H) cubes and stored in laminated plastic bags at room temperature. Each sample was compressed to 50% using a P100 probe at a crosshead speed of 2 mm/s, with 30 repetitions for each measurement.

#### 2.8.3. Environmental Scanning Electron Microscopy

The PBM samples with desirable textural properties were selected to study their structural characteristics. Images of the PBM samples were acquired using an environmental scanning electron microscope (Quattro ESEM, Thermo Scientific^TM^, Waltham, MA, USA) [47] at 50× magnification. The analysis was conducted at the National Science and Technology Development Agency (NSTDA) Characterization and Testing Service Center (NCTC), Pathum Thani, Thailand. The PBM samples were cut into small pieces and then sectioned into 10 × 10 × 5 mm (W × L × H) cross-sections to visualize the fiber structure.

### 2.9. Amino Acid Analysis

Amino acid contents in the PBM samples were analyzed by the Food Quality Assurance Service Center (FQA), Institute of Food Research and Product Development (IFRPD), Kasetsart University, Thailand, using acid hydrolysis, as described by [48]. Method 994.12, except for tryptophan. The sample was weighed, and 2 mL of 6 M HCl (Thermo scientific, USA) was added. The mixture was digested with a block heater (model SBH130D, Stuart, London, UK) at 110 °C for 24 h. After digestion, the volume was adjusted with deionized water. The amino acids were analyzed using an amino acid analyzer (model LA8080, Hitachi, Tokyo, Japan) with ion exchange resin and ninhydrin post-column derivatization (Agilent Technologies, Waldbronn, Germany). The absorbance was measured at 570 nm and 440 nm (for proline) using the ninhydrin reaction as the oxidant. Tryptophan was quantitatively analyzed using the method described by Zhaolai Dai [49]. The sample was weighed, and then 2 mL of 4M KOH was added. The mixture was digested with a block heater at 110 °C for 24 h. After digestion, the volume was adjusted with deionized water and analyzed using an HPLC analyzer (model HP 1260, Agilent Technologies, Germany).

The amino acid composition of the LM-treated PBM was used to estimate the amino acid score as milligrams of amino acid in 100 g of LM-treated PBM divided by milligrams of amino acid in the requirement pattern for Thai adults × 100 [50]. The amino acid score was calculated as a percentage of the amino acid requirement for Thai adults suggested by [51].

### 2.10. Statistical Analysis

The data obtained in this study were expressed as the mean of at least three replicates ± the standard deviation (SD). A completely randomized design (CRD) was used to analyze the data related to the shelf life study of microbial growth on the PBM samples, physicochemical characteristics, pH and TA values, color, texture, and amino acid contents. The data were analyzed using SPSS Statistics version 22.0 (IBM Corp., Armonk, NY, USA). Before analysis, the assumptions of normality and homoscedasticity of variances were examined using the Shapiro–Wilk and Levene’s tests, respectively. When both assumptions were satisfied (*p* > 0.05), means were compared by analysis of variance (ANOVA), with Duncan’s multiple range test (DMRT) performed for post hoc multiple comparisons. Statistically significant differences were recognized at *p* < 0.05.

## 3. Results

### 3.1. Number of Microorganisms in the PBM Samples

The microbiological quality of PBM under the four conditions at 4 °C for 21 days is presented in Table 1. Under the control conditions, the total plate count in PBM samples exhibited a decreasing trend from day 0 to day 14 of storage, as 6.96 to 4.76 log CFU/g, respectively. On the 21st day of storage, the total microbial count increased to 5.63 log CFU/g.

Surprisingly, no yeast or mold was detected in the four PBM samples throughout the 21-day storage period. The LAB count in the control samples increased from 2.55 log CFU/g on day 0 to 2.95 log CFU/g on day 14 and then decreased to 1.60 log CFU/g on day 21. The *Bacillus* spp. bacteria on MYP agar with pinkish cloudy colonies, detected on day 0 were 5.40 log CFU/g. No bacteria were detected in the control samples on days 7, 14, and 21 of storage, while the yellowish non-cloudy colonies recorded 4.44 and 5.06 log CFU/g on days 0 and 21, respectively. LAB-treated samples exhibited microbial counts on plate count agar that matched LAB on MRS agar throughout the 21-day storage period. Therefore, the colony counts on the PCA plates were likely LAB. The PBM samples treated with LM and LS showed non-significantly different yellowish non-cloudy colonies at 3 log CFU/g on MYP agar throughout the 21-day storage period. MALDI-TOF analysis was conducted to identify the bacterial strains on MYP agar, with *B. cereus* and *B. mojavensis* detected from the pinkish cloudy colonies on the control samples at day 0.

### 3.2. pH and TA of PBM

Table 2 presents the pH and TA of PBM samples stored at 4 °C for 21 days. The control samples exhibited significantly higher pH levels (6.77–7.01) compared to LAB (LM, LS, and PA) treated samples. The TA value in the non-treated samples, relative to lactic acid content, ranged from 0.09% to 0.22%. By contrast, LAB-treated samples demonstrated lower pH values (4.75–5.16) and higher acid contents (0.37–0.56%). The PA strain exhibited higher pH levels than the LM and LS strains. During our preliminary study, the lipid oxidation reaction substance, as measured by the thiobarbituric acid number (TBARS) of the four PBM samples, remained consistent throughout the 21 days of storage, ranging from 0.64 to 0.80 mg/kg. The TBARS values were less than 1 mg MDA/kg, generally considered the acceptable limit for rancidity [52].

### 3.3. Volatile Organic Compounds

Volatile organic compounds (VOCs) detected in all the samples included 1-hexanol, 1-nonadecene, and maltol (Table 3), with acetoin and acetic acid identified in the LAB-treated samples. 1-Hexanol, a VOC with an odor of freshly mown grass, exhibited a decreasing trend from day 0 to 21, apart from the PA-treated samples, which demonstrated an increasing trend. 1-Nonadecene was abundant in the control samples but present in lower quantities under LAB-treated conditions due to higher acidity, as evidenced by the detection of acetic acid in these samples but not in the control. Maltol, a VOC imparting a sweet flavor, remained stable in the control samples throughout the storage period, with a decreasing trend observed in the LAB-treated samples. Acetoin, a VOC with a buttery flavor, was detected in both LM and LS-treated samples.

### 3.4. Color Characteristics

The color characteristics of the PBM samples stored at 4 °C for 21 days are presented in Table 4. The PBM samples treated with LM and LS strains exhibited the highest brightness (L*) values ranging from 77.79 to 79.83, with no significant difference throughout the 21 days of storage. The PA-treated sample and the control displayed lower brightness levels, with significant differences ranging from 77.23 to 78.94 and 74.56 to 75.32, respectively.

For the a* value measurements indicating a reddish shade, the control and the LAB-treated PBM samples showed no significant differences at day 0. The red shade in the control samples significantly decreased from 0.65 to 0.87 throughout the 21 days of storage, while the a* value in the LAB-treated PBM samples increased during storage from 1.00 to 1.56. The PA-treated samples exhibited the highest tendency to develop a reddish hue, followed by LM and LS-treated samples. By contrast, the control samples displayed the least pronounced red coloration from day 7 to day 21. The b* values indicated a yellow hue, with no statistically significant differences observed in all PBM samples at day 0. The yellow hue in the control sample showed no significant difference throughout the 21 days of storage. By contrast, the b* values in the LAB-treated PBM samples decreased from day 0, ranging from 19.13 to 19.95. The yellow color values of all the samples remained within the range 19.13 to 20.90 throughout the 21-day storage period.

The PBM samples treated with all LAB strains exhibited distinct color characteristics at storage day 0, with ∆E values of more than 3, and ranging from 3.95 to 4.85. At storage day 7, all the LAB-treated samples showed ∆E values of less than 3. When extending the storage to 14–21 days, only the LM and LS-treated PBM samples showed differences in color characteristics, with ∆E values ranging from 3.74 to 4.01. The PA-treated PBM samples showed no differences in color characteristics, with ∆E values of less than 3, ranging from 2.36 to 2.94. PBM samples treated with LAB strains LM and LS exhibited distinct color characteristics at storage days 0, 14, and 21, while PBM treated with strain PA showed no significant color differences from the control samples.

### 3.5. Textural Properties

The hardness analysis results of the four PBM samples (control, LM, LS, and PA) are presented in Table 5. The control sample exhibited consistent hardness throughout the storage period, ranging from 67.34 to 75.53 N. The LAB-treated samples showed significantly higher hardness compared to the control sample. No significant differences were observed in hardness between all the LAB-treated samples on days 0, 14, and 21. The resilience values of the PBM samples varied, but remained within the range 13.88 to 16.90 throughout the storage period, while the resilience value of the LS-treated PBM sample was constant throughout storage. The control and PA-treated samples exhibited significantly different cohesiveness compared to LM and LS-treated PBM samples at day 0 of storage. After 7 to 21 days of storage, the cohesiveness values of LM, LS, and PA-treated samples remained constant, ranging from 0.41 to 0.44, and were higher than the control sample. The springiness values of the PBM samples treated with the three lactic acid bacteria and stored for 7 to 21 days remained constant at around 0.9 throughout the storage period. This finding contrasted with the control sample, which exhibited a gradual decline in springiness over the 21-day storage period. The gumminess and chewiness values of all the samples remained constant throughout the 21-day storage period, except for the LS-treated sample, which exhibited an increase in both properties over time. In terms of overall gumminess and chewiness, the control samples consistently ranked the lowest, followed by PA, LM, and LS-treated samples, respectively.

### 3.6. Microstructures of PBM

**Figure 2:**
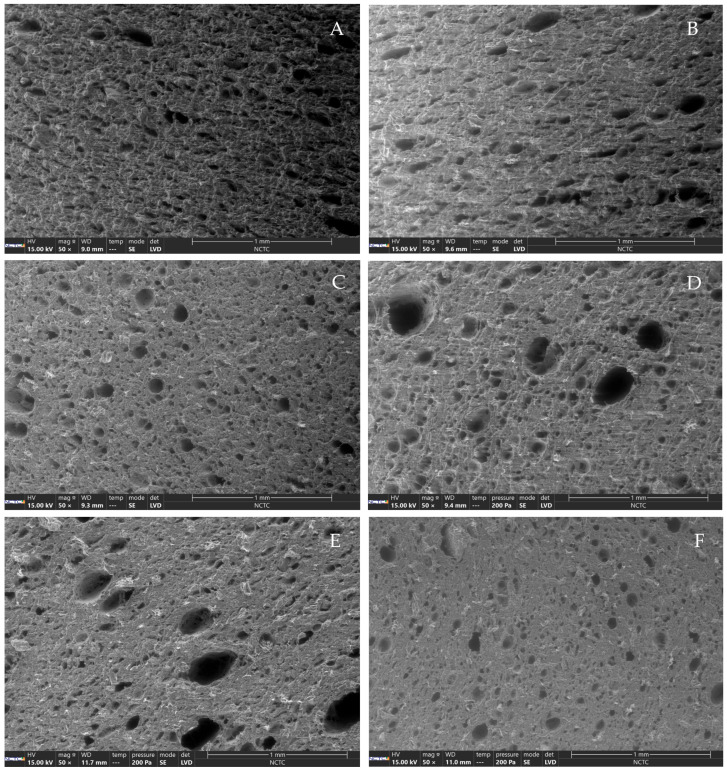
Microstructure of PBM products during storage at 4 °C under different conditions at 50× magnification; control day 0 (**A**), LM day 0 (**C**), LS day 0 (**E**), control day 21 (**B**), LM day 21 (**D**) and LS day 21 (**F**).

The LM and LS-treated PBM samples, which showed desirable textural properties, were investigated for microstructural characteristics by environmental scanning electron microscopy (ESEM). The ESEM images of the control on day 0 and day 21, as shown in Figure 2A,B, respectively, showed no noticeable differences in PBM structural morphology. By contrast, Figure 2C,D, illustrating the structural morphology of PBM-treated with LM on day 0 and day 21, revealed larger pore sizes in the LM-treated sample on day 21 compared to day 0. Figure 2E,F depict the structural morphology of PBM treated by LS on day 0 and day 21. PBM treated with LS on day 21 had smaller pore sizes of 100 µm compared to the LS-treated sample on day 0, analyzed by the WinROOF version 2015 program.

### 3.7. Amino Acid Profiles

LM and LS-treated PBM samples exhibiting favorable results for textural properties were selected for amino acid analysis. The amino acid content in the control, LM, and LS-treated PBM samples revealed predominant glutamic acid and aspartic acid (Table 6). The treated samples, particularly those inoculated with LM, exhibited a notable increase in overall amino acid content compared to the control samples. The amino acid profile of the LM-treated PMB sample showed higher amino acid content than the LS-treated PBM sample, except for glutamic acid, cystine, and methionine. The LM-treated PBM sample showed high essential amino acids compared to the control and LS-treated PBM samples. The amino acid score was further calculated according to the WHO/FAO/UNU amino acid requirements. The results in Table 7 indicated that the LM-treated PBM possessed a strong essential amino acid profile, with histidine, phenylalanine, isoleucine, and leucine demonstrating high scores compared to the WHO/FAO/UNU amino acid requirements for Thai adults [50,51].

PBM samples treated with LM and LS exhibiting favorable results from storage conditions were selected for amino acid analysis, together with the control. The LM and LS-treated PBM samples revealed predominant glutamic acid and aspartic acid (Table 6). The treated samples, particularly those inoculated with LM, exhibited increased overall amino acid content compared to the control sample. The amino acid profile of the LM-treated PMB sample mostly showed higher amino acid content than the LS-treated PBM sample, except for glutamic acid, cystine, and methionine.

## 4. Discussion

A preliminary screening for the ability of LAB to colonize fermented vegetables resulted in the isolation of 158 colonies. The identification of these isolates was confirmed at the species level by MALDI-TOF and 16S DNA sequencing, revealing seven species: *Weissella cibaria*, *P. pentosaceus*, *P. acidilactici*, *L. plantarum*, *L. pentosus*, *L. farciminis*, and *L. brevis*. From these, three strains (*L. plantarum*, *L. pentosus*, and *P. acidilactici*) were selected to enhance PBM product quality and prolong shelf life.

### 4.1. Number of Microorganisms in the PBM Samples

The absence of mold in the PBM products throughout the 21-day storage period at 4 °C was due to the low-oxygen environment that was not conducive to fungal growth. Our preliminary experiment showed low oxygen content of LAB-treated PBM in hermetically sealed nylon bags at 0.27% on day 0, which dropped to 0.10% by day 21, resulting from the airtight sealing of the nylon bags. Gorny [53] reported that an atmosphere of 3–6% O_2_ and 2–10% CO_2_ achieved microbial control and extended the shelf life of a wide variety of fresh-cut products. This result concurred with reports on fungal inhibition of fresh produce stored under modified atmospheric conditions at 5% oxygen [54,55], and this packaging condition effectively controlled fungal growth in PBM products.

The absence of *Bacillus* spp. in PBM products fermented with LAB from the beginning of storage possibly resulted from the antimicrobial activity of the LAB. Bouchibane et al. and Chadli et al. [56,57] found that *L. plantarum* and *L. pentosus* exhibited strong antimicrobial effects against *B. cereus*, with organic acid production playing a key role. The microbial count of the control sample decreased until day 14 and then increased on day 21, aligning with the number of yellowish non-cloudy colonies on MYP, which were identified as *Enterococcus faecium*. This bacterium has a minimum growth temperature at 3.6 °C [58], and the viable count in the control sample likely indicated the presence of *E. faecium*. Han et al. [59] reported that *E. faecium* was used as a probiotic and was also important from a food microbiological perspective.

### 4.2. pH and TA Values of PBM

LAB-treated PBM stored at 4 °C for 21 days recorded a decreasing pH compared to the control. Both *Lactiplantibacillus* sp. (LM and LS) showed no statistically significant differences in pH, but were statistically significantly different in TA. PA showed statistically different pH values compared with *Lactiplantibacillus* sp. LAB reduce the pH in products by fermenting carbohydrates into lactic acid, while different LAB strains produce varying levels of TA. The lower pH observed in this study resulted from lactic acid production by carbohydrate utilization of the bacterial strains. Ben-Herb et al. [28] reported that LAB strongly reduced pH due to carbohydrate utilization. Our PBM was produced in the ratio MPI:WG:DFSF as 2:3:5 (dry basis), with a total carbohydrate content of at least 56.57% from WG and DFSF according to a report by Prasert et al. [13]. Similarly, soy-based yogurt with 48% calculated carbohydrate can serve as a substrate for LAB fermentation, allowing the bacteria to produce metabolites such as lactic acid, which reduces the pH of the yogurt [60].

### 4.3. Volatile Organic Compounds

1-Hexanol was detected in soy protein PBM fermented with LAB [23]. In our study, after 21 days of storage at 4 °C, LM and LS-treated PBM products showed decreased 1-hexanol content, similar to the control samples. The PA-treated PBM sample showed increased 1-hexanol at day 21 of storage. Surprisingly, the PA-treated PBM sample showed an increase in 1-hexanol at day 21 of storage. Future analysis should explore the mechanisms underlying the effects of PA-treated PBM samples. The decrease in maltol content in all the LAB-treated samples was attributed to the pH value below 5.5. Basak and Alagha [61] explained that pH values lower than 5.5 dissolve metals from raw materials such as mung bean [62], and these dissolved metals bind with maltol compounds, leading to a subsequent decrease in maltol [63]. The odor activity values (OAVs) of specific aroma-active compounds in PBM samples were calculated as the ratio of the concentrations of aroma compounds in PBM samples to their odor detection thresholds in water [64]. The odor detection threshold values of acetoin, 1-hexanol, acetic acid, and maltol were 10.8, 0.5, 60, and 2.5 µg/g, respectively, [65]. The OAV in all PBM samples stored at day 0 and 21 were below 1, and were not considered as aroma-active compounds because their odors could not be detected by humans.

### 4.4. Color Characteristics

All the LAB-treated PBM samples showed L* values in the range 77.23 to 79.83, similar to soybean plant-based products studied by [9,36]. Their L* values (76.68) were similar to boiled chicken meat, as reported by Chiang et al. [66]. The a* values of the control sample decreased when storage time increased, whereas the a* values of the LAB-treated PBM samples increased after storage for 21 days. This product was composed of plant-based proteins, which lack the red color, and the reference formulation was also plant-protein-based with demonstrated consumer acceptance. Therefore, the authors focused on maintaining overall quality comparable to the control rather than to animal meat. The use of lactic acid bacteria resulted in an increase in the a* value, which was attributed to the production of organic acids and metabolites during fermentation that influenced pigment stability and promoted the development of a*. The b* or yellow color value of the control sample remained at 20.34–20.85 throughout storage. The LM and LS-treated PBM samples showed different color characteristics compared to the control sample at 0, 14, and 21 days of storage. The comparison with animal meat highlights an important perspective, and future research could develop coloration strategies to more closely resemble real meat.

### 4.5. Textural Properties

The textural properties as hardness, springiness, gumminess, and chewiness of the LAB-treated PBM samples were higher than the control sample, while the resilience values of all PBM products remained constant throughout storage. The LS-treated PBM sample recorded the highest hardness properties among the LAB-treated PBM samples. The increases in hardness, cohesiveness, springiness, gumminess, and chewiness in the LAB-treated samples concurred with Ou et al. and Xu et al. [23,67].

This study reported improved shelf life of PBM samples through the incorporation of LAB. PBM samples cannot be considered equivalent to animal meat, but the authors used an earlier study as a reference model to draw comparisons with previous consumer acceptance testing. LAB addition was found to alter the texture of the product, particularly the hardness, while increasing firmness. This was attributed to protein network strengthening and modifications in water-binding capacity, as also reported in studies on fermented sunflower protein concentrates [68], soy protein gels [69], and pea protein-based meat analogs [70].

### 4.6. Microstructures of PBM

The LM and LS-treated PBM samples with desirable textural properties were studied for their microstructure. The hardness, springiness, gumminess, and chewiness of LM and LS-treated PBM samples ranged from 74.59 to 111.83 N, 0.91–0.94, 31.22–49.47 N, and 28.32–45.33 N, respectively. Chiang et al. [66] reported the hardness and chewiness of extruded meat analogs with desirable textural properties as 78.61 N and 45.32 N, respectively.

The microstructure of PBM treated with LM on day 0 and day 21 (Figure 2) revealed larger pores on day 21 compared to day 0. This change was attributed to the LAB-treatment process, during which nutrients in the carbohydrate group of *L. plantarum* yielded organic acids and carbon dioxide (CO_2_) [71,72].

The microstructure of PBM treated with LS (*L. pentosus*) on day 21 showed numerous small pores, resulting in a smoother appearance than on day 0. The increasing pore size distribution was attributed to the generation of CO_2_ gas during carbohydrate fermentation by *L. pentosus*, consistent with Lipinska et al. [34], who found that lactobacilli converted carbohydrates into carbon dioxide as a secondary metabolite. The smoother appearance resulted from changes in the spherical structure of the protein due to fermentation, leading to visible multi-layer protein stacking and aggregation. The sheet structure of the protein was extended, and the protein structure expanded, with cross-links forming a cohesive whole [73]. The pH values of LM and LS-treated PBM samples on day 0 and day 21 were not significantly different, ranging from 4.85 to 4.91. Therefore, the pH of the samples was unaffected by the pores observed in the ESEM microstructural analysis. Ou et al. [23] also found that using *L. plantarum* together with *Rhodotorula mucilaginosa* and *Monascus purpureus* led to fermentative pores observed under scanning electron microscopy (SEM). Therefore, *L. plantarum* encouraged pore formation in LAB-treated PBM.

### 4.7. Amino Acid Profiles

WHO/FAO/UNU Expert Consultation [39] suggests that consumers need to consume at least two servings (150 g each) of PBM to meet daily requirements. Protein should be consumed from diverse sources [74] to receive the appropriate amount of amino acids. The LM-treated PBM sample showed higher essential amino acids compared to the control and LS-treated PBM samples. The methionine content in the LM-treated PBM sample was lower than in the LS-treated sample, while PBM samples treated with *L. plantarum* promoted the release of free amino acids [23]. The literature review showed that peeled mung beans, a component of PBM samples, had 74.5% tryptophan digestibility compared to 91.4% for chicken meat [75]. The digestibility value for mung beans was substantial, but notably less than chicken.

The amino acid score for Thai adults was calculated according to the WHO/FAO/UNU requirement pattern and the average weight of the Thai population, with tryptophan giving the lowest score. Despite individual high scores, the overall results showed that the amino acid scores of LM-treated PBM were below the total WHO/FAO/UNU amino acid requirements. The calculated amino acid score based on 100 g of LM-treated PBM showed that our product contained 47.80–99.52% of the amino acid requirements per day, with consumption of at least two servings (150 g each) of this meat substitute meeting the daily amino acid requirements.

## 5. Conclusions

PBM treated with LAB, particularly *L. plantarum*, is a potential alternative process to enhance the quality and shelf life of plant-based meat products. Our results revealed an increase in amino acid content, with no effect on VOCs in PBM treated with *L. plantarum* compared to *L. pentosus* and the non-treated sample. *L. plantarum* and *L. pentosus* improved the textural properties of PBM, with *L. plantarum*-fermented samples exhibiting a more favorable combination of increased hardness and chewiness. These findings suggest that *L. plantarum* is a promising strain for enhancing the shelf life and textural properties of PBM.

Our findings mainly improve the qualities of PBM by using LAB as a natural approach to serve the needs of clean label products, as of ultra-processed concerns. However, the limitation of this study was the incomparable appearance and texture of the real white meat. Future research could explore other alternative uses of possible single or co-culture strains that provide a plant-based meat that has a comparable appearance and texture to real white meat. In addition, the mechanisms underlying the effects of *L. plantarum* on PBM, including its impact on nutrient bioavailability and potential health benefits, should be explored. A sensory study on consumer acceptance for LAB-treated PBM would assist the food industry in further developing consumer satisfaction and sustainable products. Investigating the scalability of *L. plantarum* for industrial applications would open avenues for wider adoption in the food industry. Encapsulated or lyophilized *L. plantarum*, with no changes to its fermentation properties, would delimit its usage from the food industry perspective. Our findings highlight the potential of the LM-treatment process as a sustainable green food additive to develop clean-label PBM products.

## 6. Patents

The preservation method for plant-based meat products using lactic acid bacteria in this research study is covered by a Thai petty patent.

## Figures and Tables

**Table 1:** Number of microorganisms in PBM samples stored under different conditions for 21 days at 4 °C.

Condition	Time (Days)	Number of Microorganisms (log CFU/g)				
		Total Plate Count	Yeast and Mold	Lactic Acid Bacteria	Total MYP Count	
					Pinkish Cloudy Colonies	Yellowish Non-Cloudy Colonies
Control	0 7 14 21	6.96 ± 1.13 ^Ca^ 4.93 ± 0.16 ^Db^ 4.76 ± 1.25 ^Bb^ 5.63 ± 0.23 ^Cb^	ND ND ND ND	2.55 ± 0.20 ^Ca^ 2.38 ± 0.52 ^Ca^ 2.95 ± 0.70 ^Ba^ 1.60 ± 1.39 ^Ba^	5.40 ± 0.88 ND ND ND	4.44 ± 1.18 ^Aa^ No data No data 5.06 ± 0.31 ^Aa^
LM-treated	0 7 14 21	9.07 ± 0.11 ^Aa^ 8.85 ± 0.10 ^Ab^ 8.69 ± 0.22 ^Abc^ 8.66 ± 0.08 ^Ac^	ND ND ND ND	9.10 ± 0.14 ^Aa^ 8.90 ± 0.12 ^Aab^ 8.70 ± 0.23 ^Ab^ 8.68 ± 0.05 ^Ab^	ND ND ND ND	3.59± 0.15 ^Aa^ 3.64 ± 0.14 ^Aa^ 3.03 ± 0.88 ^Aa^ 2.90 ± 0.34 ^BCa^
LS-treated	0 7 14 21	8.62 ± 0.26 ^Ba^ 8.39 ± 0.08 ^Ca^ 8.41 ± 0.20 ^Aa^ 8.22 ± 0.14 ^Ba^	ND ND ND ND	8.62 ± 0.08 ^Ba^ 8.35 ± 0.06 ^Bb^ 8.52 ± 0.15 ^Aa^ 8.24 ± 0.06 ^Ab^	ND ND ND ND	3.79 ± 0.25 ^Aa^ 3.69 ± 0.15 ^Aa^ 3.61 ± 0.15 ^Aa^ 3.40 ± 0.35 ^Ba^
PA-treated	0 7 14 21	8.96 ± 0.15 ^ABa^ 8.62 ± 0.04 ^Ba^ 8.60 ± 0.30 ^Aa^ 8.64 ± 0.03 ^Aa^	ND ND ND ND	9.06 ± 0.20 ^Aa^ 8.67 ± 0.04 ^ABa^ 8.61 ± 0.28 ^Aa^ 8.71 ± 0.07 ^Aa^	ND ND ND ND	3.96 ± 0.40 ^Aa^ 3.74 ± 0.33 ^Aab^ 310 ± 0.14 ^Ab^ 2.37 ± 0.34 ^Cc^

Values are the mean ± standard deviation of three replicates. Different small letter superscripts within a column indicate significant differences between storage times at *p* < 0.05. Different capital letter superscripts within a column indicate significant differences between storage conditions at *p* < 0.05. ND: not detected (<1 log CFU/g); LM: *L. plantarum*; LS: *L. pentosus*; PA: *P. acidilactici*; MYP: Mannitol egg yolk polymyxin agar.

**Table 2:** pH and TA values of PBM samples stored under different conditions for 21 days at 4 °C.

Condition	Time (Days)	pH	TA (%)
Control	0 7 14 21	7.00 ± 0.02 ^Aa^ 6.90 ± 0.15 ^Aa^ 6.77 ± 0.13 ^Aa^ 7.01 ± 0.05 ^Aa^	0.22 ± 0.03 ^Ba^ 0.21 ± 0.03 ^Ca^ 0.19 ± 0.02 ^Ca^ 0.09 ± 0.01 ^Ca^
LM-treated	0 7 14 21	4.91 ± 0.11 ^Ca^ 4.90 ± 0.04 ^BCa^ 4.86 ± 0.07 ^Ca^ 4.88 ± 0.05 ^Ca^	0.53 ± 0.05 ^Aa^ 0.43 ± 0.03 ^Bb^ 0.54 ± 0.02 ^Aa^ 0.37 ± 0.03 ^Bb^
LS-treated	0 7 14 21	4.87 ± 0.08 ^Ca^ 4.84 ± 0.04 ^Ca^ 4.75 ± 0.04 ^Ca^ 4.85 ± 0.04 ^Ca^	0.51 ± 0.03 ^Aab^ 0.55 ± 0.04 ^Aa^ 0.43 ± 0.02 ^Bc^ 0.45 ± 0.04 ^Abc^
PA-treated	0 7 14 21	5.13 ± 0.08 ^Ba^ 5.12 ± 0.16 ^Ba^ 5.16 ± 0.07 ^Ba^ 5.05 ± 0.14 ^Ba^	0.56 ± 0.05 ^Aa^ 0.47 ± 0.02 ^Ba^ 0.55 ± 0.09 ^Aa^ 0.40 ± 0.05 ^ABa^

Values are the mean ± standard deviation of three replicates. Different small letter superscripts within a column indicate significant differences between storage times at *p* < 0.05. Different capital letter superscripts within a column indicate significant differences between storage conditions at *p* < 0.05. LM: *L. plantarum*; LS: *L. pentosus*; PA: *P. acidilactici.*

**Table 3:** VOC analysis of PBM samples stored under different LAB-treated conditions for 21 days at 4 °C.

Condition	Time (Days)	Relative Concentration (µg/g)				
		Acetoin (RI = 1282)	1-Hexanol (RI = 1361)	Acetic Acid (RI = 1454)	1-Nonadecene (RI > 1800)	Maltol (RI > 1800)
Control	0 21	ND ND	0.07 0.02	ND ND	3.23 3.60	0.06 0.07
LM-treated	0 21	0.02 0.01	0.02 0.01	0.14 0.07	0.28 0.08	0.03 0.01
LS-treated	0 21	ND 0.02	0.04 0.02	0.30 0.30	0.26 0.27	0.05 0.02
PA-treated	0 21	ND ND	0.06 0.11	0.06 0.17	1.89 ND	0.06 ND

ND: not detected; LM: *L. plantarum*; LS: *L. pentosus*; PA: *P. acidilactici*.

**Table 4:** Color changes in the PBM samples during storage under different conditions for 21 days at 4 °C.

Condition	Time (Days)	L*	a*	b*	∆E
Control	0 7 14 21	75.01 ± 0.25 ^Ca^ 75.32 ± 0.47 ^Ca^ 74.98 ± 0.27 ^Ca^ 74.56 ± 0.16 ^Ca^	0.90 ± 0.09 ^Aa^ 0.87 ± 0.07 ^Ca^ 0.65 ± 0.06 ^Cb^ 0.75 ± 0.13 ^Cab^	20.49 ± 0.43 ^Aa^ 20.34 ± 0.62 ^Aa^ 20.85 ± 0.23 ^Aa^ 20.81 ± 0.10 ^Aa^	- ** - - -
LM-treated	0 7 14 21	79.49 ± 0.53 ^ABa^ 77.79 ± 0.12 ^ABa^ 78.58 ± 0.56 ^Aa^ 78.42 ± 0.67 ^Aa^	0.93 ± 0.11 ^Aa^ 1.32 ± 0.17 ^Ba^ 1.00 ± 0.28 ^Ba^ 1.12 ± 0.28 ^Ba^	20.58 ± 0.25 ^Aa^ 19.80 ± 0.47 ^Aba^ 19.91 ± 0.48 ^Aa^ 19.87 ± 0.14 ^Aa^	4.48 2.57 3.74 3.99
LS-treated	0 7 14 21	79.83 ± 0.08 ^Aa^ 78.05 ± 0.08 ^Ac^ 78.58 ± 0.30 ^Ab^ 78.27 ± 0.18 ^Abc^	0.61 ± 0.08 ^Ad^ 1.33 ± 0.07 ^Ba^ 1.04 ± 0.05 ^Bc^ 1.18 ± 0.03 ^ABb^	20.90 ± 0.47 ^Aa^ 19.72 ± 0.10 ^Aba^ 19.86 ± 0.24 ^Aa^ 19.36 ± 0.83 ^Aa^	4.85 2.84 3.75 4.01
PA-treated	0 7 14 21	78.94 ± 0.28 ^Ba^ 77.23 ± 0.25 ^Bb^ 77.31 ± 0.23 ^Bb^ 77.29 ± 0.67 ^Bb^	1.05 ± 0.24 ^Ab^ 1.56 ± 0.12 ^Aa^ 1.29 ± 0.11 ^Aab^ 1.43 ± 0.15 ^Aa^	20.13 ± 0.33 ^Aa^ 19.13 ± 0.49 ^Ba^ 19.41 ± 0.78 ^Aa^ 19.95 ± 0.44 ^Aa^	3.95 2.36 2.81 2.94

Values are the mean ± standard deviation of three replicates. Different small letter superscripts within a column indicate significant differences between storage times at *p* < 0.05. Different capital letter superscripts within a column indicate significant differences between storage conditions at *p* < 0.05. ∆E values were calculated relative to the control conditions, with a value of less than 3 indicating no discernible visual difference. **: no data; LM: *L. plantarum*; LS: *L. pentosus*; PA: *P. acidilactici.*

**Table 5:** Textural properties of the PMB samples during storage under different LAB-treated conditions for 21 days at 4 °C.

Condition	Time (Days)	Hardness (N)	Resilience (%)	Cohesiveness	Springiness	Gumminess (N)	Chewiness (N)
Control	0 7 14 21	67.75 ± 6.72 ^Ba^ 75.53 ± 7.51 ^Ba^ 67.34 ± 2.51 ^Ba^ 69.71 ± 2.61 ^Ba^	15.78 ± 0.06 ^ABb^ 15.02 ± 0.55 ^Ac^ 14.76 ± 0.11 ^Ac^ 16.90 ± 0.54 ^Aa^	0.40 ± 0.01 ^BCab^ 0.39 ± 0.01 ^Cb^ 0.38 ± 0.01 ^Bb^ 0.42 ± 0.01 ^Aa^	0.90 ± 0.01 ^Ca^ 0.89 ± 0.01 ^Aa^ 0.89 ± 0.01 ^Aa^ 0.88 ± 0.01 ^Ba^	27.04 ± 2.94 ^Ba^ 29.47 ± 3.77 ^Ba^ 25.46 ± 0.86 ^Ba^ 29.13 ± 0.68 ^Ba^	24.26 ± 2.57 ^Ba^ 26.34 ± 3.27 ^Ba^ 22.55 ± 0.96 ^Ba^ 25.63 ± 0.86 ^Ca^
LM-treated	0 7 14 21	82.89 ± 13.36 ^ABa^ 74.59 ± 4.50 ^Ba^ 99.57 ± 19.75 ^Aa^ 105.07 ± 24.35 ^Aa^	16.63 ± 0.90 ^Aa^ 15.08 ± 0.55 ^Ab^ 14.87 ± 0.44 ^Ab^ 14.84 ± 0.63 ^Bb^	0.46 ± 0.01 ^Aa^ 0.42 ± 0.01 ^ABb^ 0.43 ± 0.01 ^Ab^ 0.42 ± 0.02 ^Ab^	0.94 ± 0.02 ^Aa^ 0.91 ± 0.02 ^Ab^ 0.93 ± 0.01 ^Aab^ 0.92 ± 0.01 ^Aab^	38.05 ± 6.87 ^Aa^ 31.22 ± 1.77 ^Ba^ 42.77 ± 9.23 ^Aa^ 44.15 ± 11.75 ^Aa^	35.88 ± 5.78 ^Aa^ 28.32 ± 1.61 ^Ba^ 39.65 ± 8.52 ^Aa^ 40.46 ± 10.58 ^ABa^
LS-treated	0 7 14 21	96.27 ± 1.51 ^Aa^ 99.08 ± 7.97 ^Aa^ 108.77 ± 5.49 ^Aa^ 111.83 ± 10.99 ^ABa^	15.32 ± 0.25 ^Ba^ 15.74 ± 0.25 ^Aa^ 15.36 ± 0.08 ^Aa^ 15.77 ± 0.36 ^ABa^	0.43 ± 0.01 ^Ba^ 0.44 ± 0.01 ^Aa^ 0.44 ± 0.01 ^Aa^ 0.44 ± 0.01 ^Aa^	0.92 ± 0.01 ^Ba^ 0.91 ± 0.01 ^Aa^ 0.92 ± 0.01 ^Aa^ 0.92 ± 0.01 ^Aa^	41.06 ± 1.20 ^Ac^ 43.34 ± 3.06 ^Abc^ 47.70 ± 2.49 ^Aab^ 49.47 ± 4.62 ^Aa^	37.85 ± 1.10 ^Ac^ 39.53 ± 2.55 ^Abc^ 44.06 ± 2.54 ^Aab^ 45.33 ± 3.76 ^Aa^
PA-treated	0 7 14 21	82.16 ± 10.80 ^ABa^ 77.51 ± 12.45 ^Ba^ 97.35 ± 3.95 ^Aa^ 86.52 ± 12.46 ^ABa^	13.88 ± 0.80 ^Ca^ 15.14 ± 0.37 ^Aa^ 15.77 ± 0.80 ^Aa^ 15.15 ± 0.82 ^Ba^	0.39 ± 0.03 ^Ca^ 0.41 ± 0.01 ^BCa^ 0.43 ± 0.01 ^Aa^ 0.43 ± 0.01 ^Aa^	0.91 ± 0.01 ^BCa^ 0.89 ± 0.04 ^Aa^ 0.91 ± 0.03 ^Aa^ 0.91 ± 0.01 ^Aa^	32.21 ± 6.91 ^ABa^ 33.36 ± 7.34 ^Ba^ 42.09 ± 1.43 ^Aa^ 36.69 ± 5.53 ^ABa^	29.47 ± 6.34 ^ABa^ 30.67 ± 7.13 ^Ba^ 38.52 ± 2.58 ^Aa^ 33.13 ± 4.85 ^BCa^

Values are the mean ± standard deviation of three replicates. Different small letter superscripts within a column indicate significant differences between storage times at *p* < 0.05. Different capital letter superscripts within a column indicate significant differences between storage conditions at *p* < 0.05. LM: *L. plantarum*; LS: *L. pentosus*; PA: *P. acidilactici.*

**Table 6:** Amino acid profiles of the PBM samples at day 0 of storage.

Amino Acid Profile (mg/100 g)	Control	LM-Treated	LS-Treated
Histidine	512.26 ± 53.47	551.82 ± 7.52	533.89 ± 75.01
Threonine	688.00 ± 5.76	728.52 ± 28.84	673.88 ± 28.23
Valine	854.74 ± 21.14	880.38 ± 1.76	857.05 ± 17.27
Methionine	321.74 ± 46.89	334.49 ± 39.38	340.50 ± 39.80
Phenylalanine	1078.31 ± 99.93	1127.67 ± 71.62	1092.59 ± 71.80
Isoleucine	767.58 ± 41.19	787.29 ± 24.45	761.62 ± 24.42
Leucine	1459.67 ± 63.80	1521.33 ± 27.15	1467.33 ± 27.61
Lysine	997.34 ± 34.93	1030.08 ± 33.66	989.17 ± 33.09
Tryptophan	37.96 ± 26.95	106.03 ± 86.18	98.02 ± 86.38
Aspartic acid	1858.00 ± 137.72	1906.33 ± 151.27	1819.67 ± 15.89
Glutamic acid	5260.67 ± 66.67	5610.33 ± 271.72	5265.33 ± 27.23
Serine	1055.07 ± 62.25	1153.67 ± 23.69	995.59 ± 23.69
Glycine	790.68 ± 26.05	823.80 ± 18.76	790.59 ± 18.20
Arginine	1168.00 ± 39.36	1203.33 ± 4.73	1161.67 ± 4.01
Alanine	752.18 ± 19.85	776.24 ± 12.26	765.19 ± 12.26
Tyrosine	623.71 ± 26.20	670.14 ± 11.50	655.25 ± 11.01
Cystine	434.25 ± 61.19	418.82 ± 68.11	502.76 ± 68.30
Proline	1480.67 ± 74.33	1566.67 ± 13.58	1524.00 ± 13.96

LM: *L. plantarum*; LS: *L. pentosus*; PA: *P. acidilactici.*

**Table 7:** Amino acid scores according to the WHO/FAO/UNU requirement pattern for Thai adults of the LM-treated PBM sample.

Essential Amino Acid	Requirement Pattern ^1^ (mg/kg BW per Day)	Amino Acid Requirement ^2^ (mg/Day)	Amino Acid Score LM-Treated PBM ^3^ (%)
Histidine	10	554.50	99.52
Valine	26	1441.70	61.07
Methionine	10	554.50	60.32
Phenylalanine	25	1386.25	81.35
Isoleucine	20	1109.00	70.99
Leucine	39	2162.55	70.35
Lysine	30	1663.50	61.92
Tryptophan	4	221.80	47.80

^1^ WHO/FAO/UNU [39]. Expert Consultation Report for adults. ^2^ Amino acid requirement of Thai adults [40]. ^3^ Amino acid score: (mg of amino acid in 100 g of LM-treated PBM/mg of amino acid in the requirement pattern) × 100. LM: *L. plantarum*; LS: *L. pentosus*; PA: *P. acidilactici.*

## Data Availability

The original contributions presented in this study are included in the article. Please direct all inquiries to the corresponding author.
